# Association between treatment-related lymphopenia and survival in glioblastoma patients following postoperative chemoradiotherapy

**DOI:** 10.1007/s00066-021-01855-5

**Published:** 2021-10-06

**Authors:** Roberto Mapelli, Chiara Julita, Sofia Paola Bianchi, Nicolò Gallina, Raffaella Lucchini, Martina Midulla, Flavia Puci, Jessica Saddi, Sara Trivellato, Denis Panizza, Elena De Ponti, Stefano Arcangeli

**Affiliations:** 1 Department of Radiation Oncology, University of Milan Bicocca and San Gerardo Hospital, Monza, Italy; 2 Department of Medical Physics, San Gerardo Hospital, Monza, Italy

**Keywords:** Lymphopenia, Immunosuppression, Glioblastoma, Radiotherapy, Temozolomide

## Abstract

**Purpose:**

Our study investigated the association between treatment-related lymphopenia and overall survival (OS) in a series of glioblastoma (GBM) patients. We also explored clinical and dosimetric predictors of lymphocytes depletion.

**Methods:**

Between 2015 and 2019, 64 patients were treated at the same institution with postoperative chemoradiotherapy. Peripheral lymphocyte count (PLC) data and dose–volume histogram parameters were collected. Radiotherapy (RT) schedule consisted in standard total dose of 60 Gy in 30 daily fractions, with concomitant and adjuvant temozolomide (TMZ). Posttreatment acute absolute lymphopenia (nadir AAL) was calculated as a PLC lower than 1.0 × 10^3^/mm^3^. Acute relative lymphopenia (ARL) was expressed by the nadir-PLC/baseline-PLC ratio < 0.5. Nadir-PLC was the lowest PLC registered between the end of RT and the first month of follow-up. Survival rates were estimated with Kaplan–Meier curves. Clinical and dosimetric variables related to AAL/ARL and OS were identified by univariate and multivariate analyses.

**Results:**

A total of 57 patients were eligible and included in the analyses. The median PLC was significantly decreased following chemoradiotherapy (2180/mm^3^ vs 900/mm^3^). Median OS was 16 months (range 5–55 months), with no significant difference between patients who developed nadir AAL and those who did not (16 months vs 16.5 months; *p* = 0.304). When considering ARL vs non-ARL, median OS was 14 months vs 26 months (*p* = 0.013), respectively. In multivariate Cox regression only age, sex, extent of surgery, access to adjuvant chemotherapy and brain D98% were independently associated with OS.

**Conclusion:**

Although iatrogenic immunosuppression could be associated with inferior clinical outcomes, our data show that treatment-related lymphopenia does not adversely affect GBM survival. Prospective studies are required to confirm these findings.

**Supplementary Information:**

The online version of this article (10.1007/s00066-021-01855-5) contains supplementary material, which is available to authorized users.

## Introduction

Radiation-induced lymphopenia has been associated with poor survival in several solid tumors such as, esophageal [1], lung [2–4], pancreatic [5] and cervical cancers [6, 7]. Lymphocytes are among the most radiosensitive cells in the body with a D10 (dose to reduce the total amount of surviving cells to 10% of the initial value) of around 3 Gray (Gy) only [8, 9]. It is then possible that even a low dose given in short daily intervals may interfere with the priming process of T lymphocytes and their memory functions, resulting in an immunosuppressive status potentially related to a more rapid disease progression and shorter survival.

Concurrent radiation therapy (RT) and temozolomide (TMZ) followed by adjuvant monthly TMZ after surgery remains a mainstay treatment modality for most patients with glioblastoma (GBM) [10].

These multiple therapeutic interventions, however, cause up to 40% of high-grade glioma patients to develop severe lymphopenia (grade 3–4 toxicity) within 2 months after course initiation [11]. Such a decline of the immune system, even enhanced by the combination with steroids, might result in an increased susceptibility to opportunistic infections such as pneumocystis pneumonia [12, 13] and a reactivation of cytomegalovirus, as a rare complication [14, 15]. But, more importantly, it might turn out to be associated with worse survival. A mathematical model used to estimate the RT exposure of circulating lymphocytes during RT to the brain has shown that over 95% of circulating cells received a lymphotoxic dose after a conventional course of 30 daily fractions of 2 Gy [16].

While initial preclinical experiences have revealed promising results with a wide array of immune therapies that have generated hopes for the treatment of this fatal disease [17, 18], current clinical practice is still based on the combination of RT and TMZ that can lead to significant lymphopenia in high-grade glioma patients [11, 19–21], thus negatively affecting patients’ outcomes.

The aim of this study is to investigate the correlation of lymphocyte depletion during concurrent RT/TMZ with outcomes in a cohort of patients affected by GBM, and to examine the clinical and dosimetric factors that might predict the development of acute severe lymphopenia.

## Materials and methods

### Patient population

Data of 64 patients with newly diagnosed World Health Organization (WHO) grades IV glioma treated postoperatively with RT and concurrent TMZ at a single institution, between March 2015 and July 2019, were retrospectively reviewed. Patients were excluded from the analysis in case of (a) previous irradiation to head and neck region; (b) open or stereotactic biopsy only; c) dose to the tumor area lower than 54 Gy; d) inaccessible RT dosimetric data and/or lack of information on lymphocyte count; e) follow-up shorter than 12 months. A total of 57 patients were eligible and included in the analyses; 7 patients were excluded because of lack of data on lymphocyte count.

The research was carried out according to the principles set out in the Declaration of Helsinki 1964 and all subsequent revisions. Informed consent was obtained from all individual participants and this study was reviewed and approved by the Institutional Review Board.

### Radiotherapy and chemotherapy

All patients were carefully immobilized before RT treatment with a thermoplastic head mask. Treatment simulation consisted of a CT (Computed Tomography) scan without contrast. Then, the CT images were coregistered with postoperative contrast-enhanced T1 MRI (Magnetic Resonance Imaging) sequences.

Gross tumor volume (GTV) was defined as the surgical cavity plus any residual contrast enhancement on postoperative T1 sequences. T2 FLAIR sequences were used to include edema, if deemed appropriate. A variable expansion of GTV ranging from 1 to 1.5 cm was performed in order to obtain the clinical target volume (CTV), according to patient’s anatomy. CTV was further expanded by 3–5 mm to generate a planning target volume (PTV) to account for any set-up and movement errors. Organs-at-risk (OARs) constraints were the following: maximum doses to the brainstem, optic nerves and optic chiasm ≤ 54 Gy; spinal cord ≤ 45 Gy; lens ≤ 6 Gy [22].

For 52 patients, the RT course consisted in 3D-CRT (3D Conformal Radiation Therapy) with 6 or 15 MV photons. Five patients received volumetric modulated arc therapy (VMAT) with 6 megavoltage photons. In all, 56 patients received a total radiation dose of 60 Gy, administered in 2.0 Gy daily fractions, 5 times per week. In a single case, the total dose achieved was 54 Gy in 27 fractions.

All patients were evaluated by a medical oncologist and received daily TMZ (75 mg/m^2^) for 6 consecutive weeks, beginning at the start of RT. After a 4-week break, patients received adjuvant TMZ at 150–200 mg/m^2^ for 5 consecutive days, repeated every 28 days for a maximum of 6 cycles.

### Lymphocyte evaluation

Baseline data were collected in a range between the first day of RT and 1 month before treatment beginning. Routine blood tests were obtained weekly during RT and subsequently repeated immediately at the end of RT and within 1, 6 and 12 months after treatment conclusion. The peripheral lymphocyte counts (PLCs) were measured with an automated analyzer system. Lymphopenia was graded according to the Common Terminology Criteria for Adverse Events version 5.0 (CTCAE v5.0).

### Dose–volume histogram parameters

Whole brain (WB), GTV, CTV, PTV and OARs were delineated by an expert radiation oncologist. The hypothalamic region (HR) was retrospectively delineated by an experienced neuroradiologist who was blinded to the patients’ clinical history.

The WB was contoured from the top of the skull to the foramen magnum.

Dose distributions were calculated with Monaco HD (Elekta AB, Stockholm, Sweden) treatment planning system (TPS) in 5 patients and Oncentra MasterPlan TPS (Elekta AB, Stockholm, Sweden) in 52 patients. Data about the volumetric extension of GTV, CTV, PTV, HR and WB were collected. The dosimetric parameters included: dose covering 98% (D98%) and 2% (D2%) of brain volume and mean dose (Dmean) brain; D98%, D95% and Dmean PTV; D2% and D50% brainstem; D2% optic chiasm. The following parameters were collected for the hypothalamus, although they were not used for plan evaluation: D98%, D2%, D50%, minimal dose (Dmin), maximal dose (Dmax) and Dmean.

### Follow-up and endpoints

After treatment’s completion, each patient was routinely followed at 3‑month intervals.

The primary endpoints were acute absolute lymphopenia (AAL) and acute relative lymphopenia (ARL), while the secondary endpoint was overall survival (OS). AAL was determined as a nadir-PLC below 1000 cells/mm^3^, with 4 grades of severity according to CTCAE v5.0. Namely, grade 1 (G1) corresponded to a PLC = 1000–800 cells/mm^3^; G2 = 800–500 cells/mm^3^; G3 = 500–200 cells/mm^3^; G4 < 200 cells/mm^3^. ARL was defined as the nadir-PLC/baseline-PLC ratio < 0.50. Nadir-PLC represented the lowest value of circulating lymphocytes registered within the first month of follow-up (FUP) after RT completion. OS was determined from the date of diagnosis until death from any cause; those patients alive at the time of last follow-up (7 October 2020) were censored.

### Statistical analysis

Patient baseline characteristics were summarized using descriptive statistics, and the difference between lymphopenia groups were compared using Wilcoxon test for continuous variables and Fisher exact test for categorical variables. Differences in PLCs were studied with the analysis of variance (ANOVA). Survival probability was estimated using the Kaplan–Meier method and compared between groups through log-rank statistics. A univariate Cox’s regression model was used to assess an association between potential prognostic factors and OS. To assess whether lymphopenia was an independent predictor of survival, a multivariate Cox’s regression model was constructed using other prognostic factors that had attained a *p*-value less than 0.05 in the univariate analysis. All analyses were two-sided, and significance was set at a *p*-value of 0.05. Stata software 9.0 (Stata Corporation, College Station, TX, USA) was used to perform statistical analysis.

## Results

### Basic characteristics

All 57 patients included in the study were affected by WHO grade IV glioma (Table 1). Six (10.5%, 6/57) had to withdraw TMZ before the end of treatment due to development of grade 1–2 acute thrombocytopenia, with a mean platelet count of 71,800/mm^3^. Two patients experienced grade 1 neutropenia (neutrophils = 1500–2000/mm^3^), as well. In 49 (86.0%, 49/57) cases, at least a cycle of adjuvant TMZ was administered. Dexamethasone was administered in 11 (19.3%, 11/57) patients since the beginning of RT, at a median dose of 4 mg/day (range: 0–8 mg/day). By the end of RT, 45 patients (78.9%, 45/57) received dexamethasone at a median dose of 4 mg/day (range: 0–16 mg/day). Subsequently, steroids use decreased within 1 year of follow-up (Online Resource 1).

**Table 1:** Clinical, biological and dosimetric features of the studied population

Features	
**Number of patients**	**57**
**Clinical characteristics**	
Sex male (%)	31 (54.4%)
Age^a^	61.0 (53.0–68.0)
Macroscopic radical surgery (%)	16 (28.1%)
Time between surgery and RT ≥ 6 weeks (%)	49 (86.0%)
KPS at 1st visit^a^	80 (70–80)
Time of FUP (months)^a^	16.7 (12.7–28.8)
Steroid dose at 1 month of FUP ≥ 4 mg/day (%)	29 (50.9%)
CT pre-RT (%)	34 (59.6%)
CT/RT concomitant (%)	57 (100%)
Adjuvant CT (%)	49 (86.0%)
Concomitant CT interrupted	6 (10.5%)
**Pathological characteristics**	
Grade IV WHO	57 (100%)
*IDH status*	
Mutated (%)	1 (1.8%)
Non-mutated (%)	48 (14.0%)
Unknown (%)	8 (17.2%)
*MGMT status*	
Hypermethylated (%)	22 (38.6%)
Non-hypermethylated (%)	20 (35.1%)
Unknown (%)	15 (26.3%)
*Tumor location*	
Frontal lobe (%)	16 (28.1%)
Parietal lobe (%)	5 (8.8%)
Temporal lobe (%)	26 (45.6%)
Occipital lobe (%)	7 (12.3%)
Corpus callosum—fornix (%)	2 (3.5%)
Cerebellum (%)	1 (1.8%)
**Lymphocyte count**	
PLC pre-RT^a^	2180 (1540–2710)
PLC nadir^a^	900 (730–1450)
PLC 1–6 months FUP^a^	970 (730–1400)
PLC 6–12 months FUP^a^	1470 (1100–1650)
ARL nadir (%)	29 (50.9%)
**Dosimetric characteristics**	
VMAT (%)	5 (8.8%)
GTV^a^	64.5 cm^3^ (41.6–91.7)
CTV^a^	143.0 cm^3^ (105.6–161.5)
PTV^a^	233.2 cm^3^ (174.0–259.6)
*PTV*	
Dmean^a^	60.4 Gy (60.1–60.7)
D98%^a^	57.5 Gy (56.0–58.0)
D95%^a^	58.2 Gy (57.5–58.6)
*Brainstem*	
D50%^a^	22.3 Gy (5.9–27.7)
V50^a^	8.9% (0.63–15.9)
D2%^a^	56.7 Gy (41.9–58.6)
*Chiasm*	
D2%^a^	30.4 Gy (13.0–54.9)
*Brain*	
V50^a^	22.4% (18.3–28.9)
Dmean^a^	25.2 Gy (21.4–29.1)
D98%^a^	1.2 Gy (0.8–1.4)
D2%^a^	61.8 Gy (61.4–62.3)
*Hypothalamus*	
Volume^a^	7.7 cm^3^ (6.8–8.9)
D98%^a^	16.9 Gy (3.9–24.4)
D2%^a^	55.3 Gy (30.3–58.7)
D50%^a^	25.9 Gy (14.0–38.3)
Dmin^a^	13.2 Gy (3.2–23.1)
Dmax^a^	57.9 Gy (39.2–59.5)
Dmean^a^	28.8 Gy (18.6–41.3)

*RT* radiotherapy, *CT* chemotherapy, *FUP* follow-up, *KPS* Karnofsky performance status, *MGMT* O6-methylguanine-DNA-methyltransferase, *IDH* isocitrate dehydrogenase, *PLC* peripheral lymphocyte count, *ARL* acute relative lymphopenia, *VMAT* volumetric modulated arc therapy, *GTV* gross tumor volume, *CTV* clinical target volume, *PTV* planning target volume, *Dmax* maximal dose, *Dmean* mean dose, *Dmin* minimal dose, *D98%* dose administered to 98% of volume, *D95%* dose administered to 95% of volume, *D50%* dose administered to 50% of volume, *D2%* dose administered to 2% of volume, *V50* volume that received 50 Gy, *cm*^*3*^ cubic centimeter, *Gy* Gray

^a^Median value (I–III quartile)

During the period of observation, PLC underwent a marked reduction, with a median nadir of 900 cells/mm^3^ (range 130–3470 cells/mm^3^). The median baseline count was 2180 cells/mm^3^ with an interval ranging from 690 to 4520 cells/mm^3^. The reduction was highly significant at ANOVA test (*p*-value < 0.001).

**Fig. 1:**
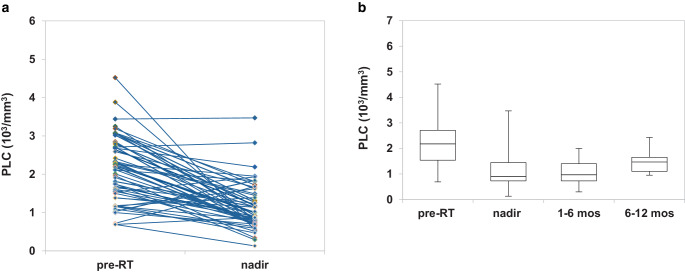
Lymphocyte count’s variation from baseline to nadir, with the onset of acute lymphopenia (**a**), lymphocyte count’s variation from baseline to 1‑year follow-up (**b**). *RT* radiotherapy, *mos* months, *PLC* peripheral lymphocyte count

Nadir AAL was observed in 31 (54.4%, 31/57) patients. Mostly, it consisted in G1–G2 lymphopenia, with only 4 (7.0%, 4/57) patients presenting grade 3 or 4 (Fig. 1a). Afterwards, the lymphocyte count kept stable during the 12-months follow-up (Fig. 1b). Considering nadir ARL, 29 (50.8%, 29/57) patients lost at least 50% of lymphocytes compared to baseline.

**Fig. 2:**
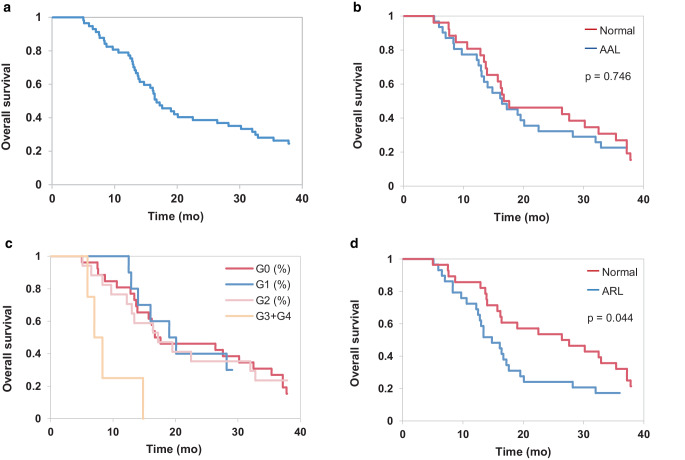
Kaplan–Meier overall survival curves of the entire group of patients (**a**), according to the presence of AAL at nadir (**b**), stratified on grades of AAL (*p* = 0.004, **c**) and according to the presence of ARL at nadir (**d**). Log-rank test, *p* value < 0.05. *AAL* acute absolute lymphopenia, *ARL* acute relative lymphopenia, *mo* month, *PLC* peripheral lymphocyte count, *G0* grade 0 (PLC > 1000 cells/mm^3^), *G1* grade 1 (PLC: 1000–800 cells/mm^3^), *G2* grade 2 (PLC: 800–500 cells/mm^3^), *G3* grade 3 (PLC: 500–200 cells/mm^3^), *G4* grade 4 (PLC < 200 cells/mm^3^)

Forty-six (80.7%, 46/57) patients died during the follow-up. Median OS was 16 months (range 5–55 months), with no significant difference between patients who developed nadir AAL and those who did not (16 months vs 16.5 months; *p*-value 0.304, respectively). The 18-months OS rate was 45.6% (26/57) for the entire population (Fig. 2a), with no significant difference between the former and the latter group (45.2%, 14/31, vs 46.2%, 12/26; *p*-value: 0.576, respectively). However, median OS was 14 months vs 26 months (*p*-value: 0.013) in ARL vs non-ARL groups. Likewise, the 18-months OS rate was 31.0% (8/29) vs 60.7% (17/28), respectively (*p*-value: 0.012).

### Acute lymphopenia and survival

Kaplan–Meier curves did not show a significant difference in survival between AAL and non-AAL patients (Fig. 2b). There were no statistical differences in stratified data as well (Fig. 2c), except for patients who developed high-grade lymphopenia (G3 + G4 group), whose number was limited (no.: 4). Considering nadir ARL, OS was markedly shorter in patients who lost > 50% of lymphocytes (Fig. 2d).

In order to investigate clinical and dosimetric factors associated with OS, univariate and multivariate Cox’s regressions were performed (Table 2). Some of the most important variables related to survival were included in the analysis. Interruption of concomitant chemotherapy (CT) was considered in order to investigate if the onset of hematological toxicities could have affected OS. MGMT hypermethylation was not added because of lack of data, as indicated in Table 1. Only variables statistically significant at the univariate analysis were included in the multivariate model. Older age, sex (male), subtotal resection of glioblastoma and higher brain D98% were independently associated with worse OS. Conversely, access to adjuvant CT was related to a better prognosis. Despite reaching statistical significance at univariate analysis, nadir ARL and G3–G4 lymphopenia were not independently associated with OS. The hypothalamic dosimetric factors were not involved in differences in survival.

**Table 2:** Cox’s regression analysis about clinical and dosimetric variables associated with overall survival

	Univariate Cox’s regression		Multivariate Cox’s regression	
Variables	HR (95% CI)	*p*-value	HR (95% CI)	*p*-value
Age	1.04 (1.01–1.07)	*0.009	1.06 (1.02–1.09)	*0.003
Sex (male vs female)	2.04 (1.12–3.70)	*0.020	3.04 (1.50–6.14)	*0.002
Extent of surgery (subtotal resection vs gross total resection)	2.35 (1.19–4.62)	*0.013	2.47 (1.08–5.65)	*0.032
Time b/w surgery and RT ≥ 6 weeks	0.79 (0.33–1.89)	0.597	–	–
Steroid dose 1‑month FUP ≥ 4 mg/day	1.88 (1.03–3.42)	*0.040	1.01 (0.50–2.04)	0.986
KPS pre-RT ≤ 70	2.04 (1.10–3.79)	*0.023	0.58 (0.25–1.38)	0.222
Concomitant CT interrupted (yes vs no)	2.62 (0.99–6.94)	0.052	–	–
Adjuvant CT (yes vs no)	0.10 (0.04–0.26)	*<0.001	0.10 (0.02–0.52)	*0.006
ARL (yes vs no)	1.82 (1.01–3.29)	*0.050	1.00 (0.47–2.13)	0.992
Lymphocytes at 1‑month FUP < 500 (G3–G4 lymphopenia)	6.02 (2.01–18.02)	*0.001	0.65 (0.13–3.42)	0.615
Radiation technique (VMAT vs 3D-CRT)	1.09 (0.33–3.58)	0.881	–	–
PTV	1.00 (0.99–1.01)	0.288	–	–
Brain Dmean	1.04 (0.99–1.10)	0.137	–	–
Brain D98%	1.99 (1.15–3.44)	*0.014	2.63 (1.38–5.03)	*0.003
Hypothalamus Dmin	1.01 (0.99–1.03)	0.332	–	–
Hypothalamus Dmax	1.00 (0.98–1.02)	0.663	–	–
Hypothalamus Dmean	1.00 (0.99–1.03)	0.538	–	–

*HR* hazard ratio, *CI* confidence interval, *b/w* between, *RT* radiotherapy, *FUP* follow-up, *KPS* Karnofsky performance status, *CT* chemotherapy, *ARL* acute relative lymphopenia, *G3* grade 3, *G4* grade 4, *VMAT* volumetric modulated arc therapy, *3D-CRT* three-dimension conformal radiotherapy, *PTV* planning target volume, *Dmean* mean dose, *D98%* dose administered to 98% of volume, *Dmin* minimal dose, *Dmax* maximal dose

^*^Variables statistically significant (*p* value ≤ 0.05)

### Acute lymphopenia and etiological factors

Association analyses between nadir AAL and major clinical and dosimetric variables were performed (Online Resource 2). Steroid dose ≥ 4 mg/day, CTV, PTV, brain V50, brain D2% were significantly associated with AAL. Smaller doses of dexamethasone and D2% brain, as well as smaller CTV and PTV volumes and brain V50, turned out to be associated with the development of AAL. All hypothalamic variables failed in reaching statistical significance.

As shown in Table 3, only the interruption of concomitant CT was significantly associated with development of nadir ARL.

**Table 3:** Clinical, biological and dosimetric factors associated with the development of ARL at nadir

Features	ARL	Non-ARL	*p*-value
Number of patients	29	28	–
**Clinical characteristics**			
Sex male (%)	16 (55.2%)	15 (53.6%)	0.557^c^
Age^a^	61 (58.7–67.0)	60.5 (52.3–64.7)	0.237^d^
Macroscopic radical surgery (%)	7 (24.1%)	9 (32.1%)	0.777^c^
Time b/w surgery and RT ≥ 6 weeks (%)	26 (89.7%)	23 (82.1%)	0.333^c^
Steroid dose 1‑month FUP ≥ 4 mg/day (%)	15 (51.7%)	14 (50.0%)	0.554^c^
CT pre-RT (%)	19 (65.5%)	15 (53.6%)	0.258^c^
CT/RT concomitant (%)	29 (100%)	28 (100%)	NA
Concomitant CT interrupted	6 (20.7%)	0 (0%)	*0.013^c^
**Pathological characteristics**			
MGMT: hypermethylated (%)	11 (57.9%)	11 (47.8%)	0.367^c^
MGMT: non-hypermethylated (%)	8 (42.1%)	12 (52.2%)	–
**Dosimetric characteristics**			
VMAT (%)	3 (10.3%)	2 (7.1%)	0.517^c^
GTV^b^	51.4 cm^3^ (41.8–90.0)	69.6 cm^3^ (60.9–80.7)	0.394^d^
CTV^b^	122.1 cm^3^ (104.8–154.1)	148.3 cm^3^ (130.7–161.4)	0.129^d^
PTV^b^	198.5 cm^3^ (174.0–243.7)	204.8 cm^3^ (155.7–223.8)	0.140^d^
*PTV*			
Dmean^b^	60.5 Gy (60.3–60.7)	60.3 Gy (60.1–60.6)	0.288^d^
D98%^b^	57.4 Gy (56.5–57.9)	57.5 Gy (56.6–57.8)	0.714^d^
D95%^b^	58.3 Gy (58.0–58.6)	58.1 Gy (57.8–58.5)	0.416^d^
*OARs*			
D50% brainstem^b^	25.3 Gy (17.9–27.3)	17.5 Gy (6.8–25.5)	0.170^d^
V50 brainstem^b^	10.6% (1.6–15.6)	6.9% (0.4–17.0)	0.785^d^
D2% brainstem^b^	57.1 Gy (51.8–57.9)	56.3 Gy (47.5–58.7)	0.867^d^
D2% chiasm^b^	39.7 Gy (27.6–53.4)	23.5 Gy (8.4–49.2)	0.161^d^
*Brain*			
V50^b^	21.7% (17.1–27.1)	24.3% (20.3–31.3)	0.135^d^
Dmean^b^	25.2 Gy (21.6–27.8)	24.5 Gy (23.1–30.1)	0.566^d^
D98%^b^	1.3 Gy (0.9–1.4)	1.0 Gy (0.8–1.3)	0.149^d^
D2%^b^	61.8 Gy (61.5–62.1)	61.9 Gy (61.7–62.0)	0.844^d^
*Hypothalamus*			
Volume^b^	7.9 cm^3^ (7.1–8.8)	8.7 cm^3^ (8.0–10.5)	0.658^d^
D98%^b^	21.9 Gy (9.8–23.9)	9.3 Gy (4.0–23.8)	0.232^d^
D2%^b^	53.6 Gy (41.0–57.6)	56.2 Gy (29.4–58.4)	0.781^d^
D50%^b^	25.9 Gy (24.0–33.7)	26.7 Gy (14.7–35.9)	0.658^d^
Dmin^b^	19.6 Gy (6.0–23.2)	6.3 Gy (3.1–21.3)	0.124^d^
Dmax^b^	57.0 Gy (46.0–58.6)	58.4 Gy (46.2–59.5)	0.706^d^
Dmean^b^	27.7 Gy (23.2–36.8)	29.6 Gy (19.1–39.0)	0.646^d^

*ARL* acute relative lymphopenia, *MGMT* O^6^-methylguanine-DNA-methyltransferase, *RT* radiotherapy, *CT* chemotherapy, *FUP* follow-up, *GTV* gross tumor volume, *CTV* clinical target volume, *PTV* planning target volume, *OARs* organs at risk, *VMAT* volumetric modulated arc therapy, *Dmax* maximal dose, *Dmean* mean dose, *Dmin* minimal dose, *D98%* dose administered to 98% of volume, *D95%* dose administered to 95% of volume, *D50%* dose administered to 50% of volume, *D2%* dose administered to 2% of volume, *V50* volume that received 50 Gy, *cm*^*3*^ cubic centimeter, *Gy* gray, *b/w* between

^a^Median value (95% interval of confidence)

^b^Median value (I–III quartile)

^c^Fisher’s exact test

^d^Wilcoxon sum rank test

*Variables statistically significant (*p* value < 0.05)

## Discussion

During the past decade, scientific evidence has emerged showing that ionizing radiations may have systemic effects converting the tumor into an individualized in situ vaccine sometimes resulting in the so-called abscopal effect, an out-of-the-field effect of local RT which can effectively immunize the patient against the irradiated tumor [23, 24]. However, as circulating lymphocyte populations are highly radiosensitive and can undergo apoptosis or depletion due to radiation exposure, this positive impact is often counteracted by radiation-induced lymphopenia, which suppresses antitumor immunity and is associated with inferior survival in patients with various solid tumors [1–7].

In the present study, we have investigated the correlation between lymphocytes depletion and survival in a cohort of patients with glioblastoma (GBM) who underwent concurrent RT/TMZ and explored possible risk factors for posttreatment acute lymphopenia.

With a median follow up of 16.7 months (I–III quartile: 12.7–28.8 months), median OS was 16 months (5–55 months) and the 18-month OS rate was 45.6% (Fig. 2a), which largely paralleled the outcomes observed in contemporary series of RT plus concomitant and adjuvant TMZ for GBM [25].

The paired analysis of pre- and posttreatment complete blood counts revealed that patients experienced a substantial reduction in median values of peripheral lymphocyte count (PLC), from 2180 cells/mm^3^ at baseline to 900 cells/mm^3^ at nadir (*p*-value < 0.001), in accordance with other studies [11, 16, 26]. Unlike others [11, 26, 27], however, when we looked at a possible impact of such treatment-related lymphopenia, log-rank test failed to identify a correlation between nadir AAL and OS (Fig. 2b). This data might be explained by the low incidence of severe AAL in this cohort. Although a significant lymphocytes depletion occurred at the treatment completion, only a minority of patients (7.0%, 4/57) experienced a G3–G4 lymphopenia (Fig. 1a). Therefore, it is unsurprising that a mild nadir AAL did not translate into a significant survival detriment. Conversely, focusing the analysis on nadir ARL, expressed by the nadir-PLC/baseline-PLC ratio < 0.50, a statistically significant correlation with OS was found in patients who lost > 50% of lymphocytes from the baseline (*p*-value = 0.044; Fig. 2d). These findings are similar to those obtained from a post hoc analysis of a randomized controlled trial of postmastectomy hypofractionated radiation therapy [28] and are among the few ones that aimed at the nadir-PLC/baseline-PLC ratio as a potential prognostic factor. Rather than AAL, the use of ARL better conveys the rapid fall off in PLC, which might be associated with poorer outcomes. Failure to complete the concomitant TMZ has emerged as a significant risk factor in the development of nadir ARL, suggesting that chemotherapy-related myelosuppression might have played a role in negatively affecting the nadir-PLC/baseline-PLC ratio.

We also investigated whether nadir ARL and severe AAL could be independently associated with OS. Despite being statistically significant at univariate Cox’s regression, both factors were not confirmed to have a prognostic value at multivariate analysis (Table 2). However, older age, gender (male), subtotal resection of glioblastoma and higher brain D98% were associated with worse OS. Notably, access to adjuvant chemotherapy (CT) after chemoradiation was related to a better prognosis. This finding was consistent with the established knowledge that adjuvant temozolomide significantly increases OS in glioblastoma patients [10]. Despite the interruption of concomitant CT being associated with the development of ARL, it did not affect OS.

As a series of preclinical studies has shown that the hypothalamus (HT) acts as an immunoregulatory center [29, 30] playing a crucial role in the regulation of peripheral immune functions, we have included it among the dosimetric factors that, together with other clinical variables, might have been potentially implied in the development of posttreatment lymphopenia. Unexpectedly, the associations analyses revealed that smaller doses of dexamethasone and brain D2%, as well as smaller CTV and PTV volumes and brain V50, turned out to be associated with the development of nadir AAL (Online Resource 2). HT dosimetric features (D98%, D2%, D50%, Dmin, Dmax, Dmean) were also not found to be independently associated with the development of treatment-related lymphopenia. These findings are not in keeping with previous studies [11, 16, 26, 31], likely owing to the scarce uniformity in the radiation techniques and volumes, and the relatively small population. However, as in patients with abnormal (< 1000 cell/mm^3^) posttreatment PLC, the most common tumor locations were the temporal and parietal lobes (58.1% in AAL vs 30.8% in non-AAL), both supplied by the middle cerebral artery, it is conceivable that the irradiation of brain areas supplied by the largest branch of the internal carotid [32] might have resulted in a more relevant PLC depletion, thus overcoming the effect of other factors.

Radiation-induced lymphopenia is commonly correlated with lower dose ranges [3, 22] because of the high radiosensitivity of lymphocytes. Commercially available TPS are usually not designed to model dose distribution of very small doses due to computational requirements and little relevance for routine treatment planning. The accuracy of calculated out-of-field doses receives less attention because they are significantly lower compared to the prescribed target dose. Several investigations have previously been devoted to the study of the out-of-field doses [33–38]. The conclusions varied widely and depended upon the treatment modalities and anatomical location of target volumes, but poor accuracy and larger underestimation of out-field dose calculations were generally highlighted. Accordingly, small doses that could affect lymphopenia may not be calculated properly using the clinical TPS. For these reasons, they were overlooked in our study. However, it cannot be excluded that the exposure of healthy tissue to integral whole-body doses, an established dosimetric factor which differentiates the dose delivery methods [39] might play a role in the iatrogenic lymphopenia, thus explaining the contradictory findings of reduced lymphopenia with smaller treatment volumes.

Beyond its intrinsic retrospective nature, our study has further limitations: we did not consider that, apart from direct toxicity, RT might have indirectly affected PLC by means of cytokine modulation [40]. For example, interleukin‑7 (IL-7), a cytokine involved in T‑cell proliferation, is well recognized to play a pivotal role in maintaining circulating T‑cell homeostasis. Furthermore, we have considered PLC as a whole entity, without discriminating different lymphocyte subpopulations that might exert their effector mechanisms after RT at different levels [41]. Finally, although MGMT-methylation status is nowadays an established prognostic factor, we could not evaluate it because it was not systematically included in the pathological assessment of resected GBM, especially in patients treated before 2017.

In conclusion, despite the evidence that immunosuppression induced by CT-RT (concomitant chemo-radiotherapy) is associated with inferior clinical outcomes, our data show that treatment-related lymphopenia does not adversely affect the prognosis in patients with GBM treated with RT/TMZ.

The use of ARL coupled with AAL is merely hypothesis-generating and must be proven in a confirmatory study. Nevertheless, an improved understanding of the biology behind AAL/ARL might help to prevent inadvertent lymphocyte depletion or to restore lymphocytes during or after CT-RT.

## Supplementary Information

Online Resource 1: Fig. 1**: **Box plot illustrating time-correlated dexamethasone dose variations. Patients’ number is indicated in brackets.

Online Resource 2**: **Clinical, biological and dosimetric factors associated with the development of AAL at nadir.
