# Supplementary material for: Association between treatment-related lymphopenia and survival in glioblastoma patients following postoperative chemoradiotherapy
Source: Strahlenther Onkol. 2021 Oct 6;198(5):448–57. doi: 10.1007/s00066-021-01855-5 (PMC9038819; doi:10.1007/s00066-021-01855-5)
Supplement: Supplementary file 1 — Online Resource 1: Fig. 1: Box plot illustrating time-correlated dexamethasone dose variations. Patients’ number is indicated in brackets. [file 66_2021_1855_MOESM1_ESM.pdf]

Acute relative lymphopenia is associated with poor survival in glioblastoma patients following postoperative chemo-radiotherapy

Roberto Mapelli[1], Chiara Julita[1], Sofia Paola Bianchi[1], Nicolò Gallina[1], Raffaella Lucchini[1], Martina Midulla[1], Flavia Puci[1], Jessica Saddi [1], Sara Trivellato[2], Denis Panizza[2], Elena De Ponti[2], Stefano Arcangeli (ORCID id: https://orcid.org/0000-0003-3880-8876)[1]

[1] Department of Radiation Oncology, University of Milan Bicocca and San Gerardo Hospital – Monza (Italy)

[2] Department of Medical Physics, San Gerardo Hospital – Monza (Italy)

Strahlentherapie und Onkologie

Corresponding author: Roberto Mapelli (ORCID id: https://orcid.org/0000-0003-1530-3138; e-mail address: r.mapelli.95@gmail.com; r.mapelli4@campus.unimib.it)

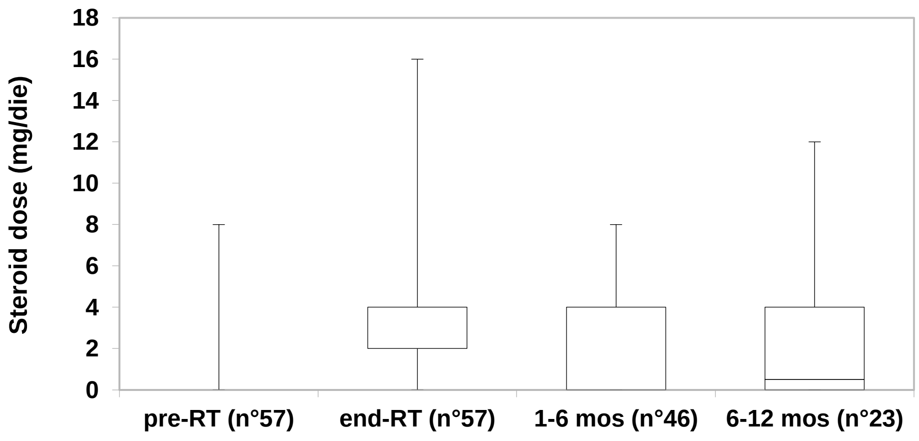

Caption: Fig. 1 Box plot illustrating time-correlated dexamethasone dose variations. Patients' number is indicated in brackets. Abbreviations: RT, radiotherapy; mos, months; mg/die, milligrams per day

Graphic program used: Microsoft Excel (Microsoft Office 365)
