# Supplementary material for: Association between treatment-related lymphopenia and survival in glioblastoma patients following postoperative chemoradiotherapy
Source: Strahlenther Onkol. 2021 Oct 6;198(5):448–57. doi: 10.1007/s00066-021-01855-5 (PMC9038819; doi:10.1007/s00066-021-01855-5)
Supplement: Supplementary file 2 — Online Resource 2: Clinical, biological and dosimetric factors associated with the development of AAL at nadir. [file 66_2021_1855_MOESM2_ESM.pdf]

# Acute relative lymphopenia is associated with poor survival in glioblastoma patients following postoperative chemo-radiotherapy

Roberto Mapelli[1], Chiara Julita[1], Sofia Paola Bianchi[1], Nicolò Gallina[1], Raffaella Lucchini[1], Martina Midulla[1], Flavia Puci[1], Jessica Saddi [1], Sara Trivellato[2], Denis Panizza[2], Elena De Ponti[2], Stefano Arcangeli (ORCID id: https://orcid.org/0000-0003-3880-8876 )[1]

[1] Department of Radiation Oncology, University of Milan Bicocca and San Gerardo Hospital – Monza (Italy)

[2] Department of Medical Physics, San Gerardo Hospital – Monza (Italy)

*Strahlentherapie und Onkologie*

*Corresponding author: Roberto Mapelli (ORCID id: https://orcid.org/0000-0003-1530-3138; e-mail address: r.mapelli.95@gmail.com; r.mapelli4@campus.unimib.it)*

| Features | AAL | Non-AAL | p-value |
|---|---|---|---|
| ***Number of patients*** | *31* | *26* | |
| ***Clinical characteristics*** | | | |
| *Sex male (%)* | *15 (48.4%)* | *16 (61.5%)* | *0.234[c]* |
| *Age [a]* | *62 (54.0 – 68.0)* | *59 (51.0– 69.3)* | *0.395[d]* |
| *Macroscopic radical surgery (%)* | *8 (25.8%)* | *8 (30.8%)* | *0.615[c]* |
| *Time b/w surgery and RT ≥ 6 weeks (%)* | *26 (83.9%)* | *23 (88.5%)* | *0.458[c]* |
| *Steroid dose 1-month FUP ≥ 4 mg/die (%)* | *12 (38.7%)* | *17 (65.4%)* | **0.040[c]* |
| *CT pre-RT (%)* | *18 (58.1%)* | *16 (61.5%)* | *0.503[c]* |
| *CT/RT concomitant (%)* | *31 (100%)* | *26 (100%)* | *NA* |
| *Concomitant CT interrupted* | *5 (16.1%)* | *1 (3.8%)* | *0.142[c]* |
| ***Pathological characteristics*** | | | |
| *MGMT: hypermethylated (%)* | *13 (52.0%)* | *9 (52.9%)* | *0.601[c]* |
| *MGMT: non-hypermethylated (%)* | *12 (48.0%)* | *8 (47.1%)* | |
| ***Dosimetric characteristics*** | | | |
| *VMAT (%)* | *3 (9.7%)* | *2 (7.7%)* | *0.585[c]* |
| *GTV [b]* | *62.9 $cm^3$ (39.7-83.0)* | *74.2 $cm^3$ (41.4-101.2)* | *0.202[d]* |
| *CTV [b]* | *124.1 $cm^3$ (98.4-157.5)* | *154.1 $cm^3$ (123.2-190.6)* | **0.034[d]* |
| *PTV [b]* | *209.3 $cm^3$ (172.9-248.8)* | *241.3 $cm^3$ (197.5-294.5)* | **0.038[d]* |
| PTV | | | |
| *Dmean [b]* | *60.3 Gy (60.1-60.6)* | *60.5 Gy (60.1-60.8)* | *0.223[d]* |
| *D98% [b]* | *57.4 Gy (56.5-58.1)* | *57.5 Gy (55.3-57.9)* | *0.553[d]* |
| *D95% [b]* | *58.3 Gy (57.7-58.8)* | *58.1 Gy (57.1-58.6)* | *0.414[d]* |
| OARs | | | |
| *D50% brainstem [b]* | *25.3 Gy (13.2-27.5)* | *14.2 Gy (4.3-28.7)* | *0.298[d]* |
| *V50 brainstem [b]* | *8.9% (1.6-15.9)* | *7.6% (0.4-14.9)* | *0.803[d]* |
| *D2% brainstem [b]* | *57.2 Gy (45.8-58.8)* | *56.3 Gy (40.0-58.1)* | *0.476[d]* |
| *D2% chiasm [b]* | *35.5 Gy (18.0-55.1)* | *26.3 Gy (7.4-54.9)* | *0.415[d]* |
| Brain | | | |
| *V50 [b]* | *21.6% (16.9-25.4)* | *25.5% (20.1-32.9)* | **0.025[d]* |
| *Dmean [b]* | *25.2 Gy (20.6-27.3)* | *26.6 Gy (21.2-31.1)* | *0.173[d]* |
| *D98% [b]* | *1.1 Gy (0.7-1.4)* | *1.3 Gy (0.8-1.7)* | *0.286[d]* |
| *D2% [b]* | *61.7 Gy (61.2-61.9)* | *62.0 Gy (61.6-62.5)* | **0.021[d]* |
| Hypothalamus | | | |
| *Volume [b]* | *7.5 $cm^3$ (6.6-8.8)* | *8.1 $cm^3$ (7.2-9.2)* | *0.168[d]* |
| *D98% [b]* | *19.1 Gy (3.1-26.0)* | *10.0 Gy (4.0-24.4)* | *0.718[d]* |

| | | | |
|---|---|---|---|
| *D2%* [b] | *53.7 Gy (32.8-59.1)* | *55.8 Gy (28.3-58.1)* | *0.622*[d] |
| *D50%* [b] | *25.9 Gy (11.2-40.1)* | *26.7 Gy (15.3-41.8)* | *0.882*[d] |
| *Dmin* [b] | *17.0 Gy (2.8-24.6)* | *8.6 Gy (3.2-21.9)* | *0.522*[d] |
| *Dmax* [b] | *57.4 Gy (42.7-59.7)* | *58.3 Gy (33.6-59.4)* | *0.718*[d] |
| *Dmean* [b] | *26.6 Gy (17.9-42.1)* | *29.6 Gy (17.4-41.7)* | *0.908*[d] |

**Online Resource 2** Clinical, biological and dosimetric factors associated with the development of AAL at nadir.

Abbreviations: AAL, acute absolute lymphopenia (< 1000 cells/mm$^3$); CI, confidence interval, MGMT, O6-methylguanine-DNA-methyltransferase; RT, radiotherapy; CT, chemotherapy; FUP, follow-up; GTV, gross tumor volume; CTV, clinical target volume; PTV, planning target volume; VMAT, volumetric modulated arc therapy; Dmax, maximal dose; Dmean, mean dose; Dmin, minimal dose; D98%, dose administered to 98% of volume; D95%, dose administered to 95% of volume; D50%, dose administered to 50% of volume; D2%, dose administered to 2% of volume; V50, volume that received 50Gy; cm$^3$, cubic centimeter; Gy, gray; b/w, between

[a] Median value (95% interval of confidence)

[b] Median value (I – III quartile)

[c] Fisher's exact test

[d] Wilcoxon sum rank test

* Variables statistically significant (p value < 0.05)
